# Exploring the association between Th17 pathway gene polymorphisms and pulmonary tuberculosis

**DOI:** 10.3389/fimmu.2022.994247

**Published:** 2022-11-21

**Authors:** Hong-Miao Li, Li-Jun Wang, Qian Huang, Hai-Feng Pan, Tian-Ping Zhang

**Affiliations:** ^1^ Department of Epidemiology and Biostatistics, School of Public Health, Anhui Medical University, Hefei, Anhui, China; ^2^ Department of Infectious Diseases, The First Affiliated Hospital of Anhui Medical University, Hefei, Anhui, China; ^3^ Department of Public Health, Medical Department, Qinghai University, Xining, China; ^4^ Department of Rheumatology and Immunology, The First Affiliated Hospital of USTC, Division of Life Sciences and Medicine, University of Science and Technology of China, Hefei, Anhui, China

**Keywords:** pulmonary tuberculosis, Th17 pathway genes, *IL-17A*, *IL-17F*, *IL-21*, *IL-23R*, single nucleotide polymorphisms

## Abstract

Th17 cells play a key role in immunity against *Mycobacterium tuberculosis* (MTB), and this study aimed to explore the association of Th17 pathway gene polymorphisms with pulmonary tuberculosis (PTB) susceptibility in a Chinese population. A total of 10 single nucleotide polymorphisms in Th17 pathway genes (*IL-17A* gene rs2275913, rs3748067, rs8193036, rs3819024, *IL-17F* gene rs7741835, rs763780, *IL-21* gene rs907715, rs2055979, *IL-23R* gene rs11805303, and rs7518660) were genotyped in 456 PTB patients and 466 controls using SNPscan technique. The *IL-23R* rs11805303 CC genotype, C allele frequencies were significantly lower in PTB patients than in controls, and the rs11805303 variant was significantly associated with the reduced risk of PTB in a recessive model. There were no significant associations between *IL-17A*, *IL-17F*, and *IL-21* gene variations and PTB risk. In *IL-17A* gene, rs2275913, rs3748067, and rs3819024 variants were associated with drug resistance in PTB patients. In *IL-17F* gene, rs7741835 variant affected drug resistance, and rs763780 variant was associated with hypoproteinemia in PTB patients. In addition, the lower frequencies of the TT genotype, T allele of rs2055979 were found in PTB patients with drug-induced liver injury. Haplotype analysis showed that *IL-23R* CG haplotype frequency was significantly lower in PTB patients than in controls, while the TG haplotype frequency was higher. In conclusion, *IL-23R* rs11805303 polymorphism may contribute to the genetic underpinnings of PTB in the Chinese population, and the *IL-17A*, *IL-17F*, and *IL-21* genetic variations are associated with several clinical manifestations of PTB patients.

## Introduction

Tuberculosis (TB) is a common chronic infectious disease caused by *Mycobacterium tuberculosis* (MTB) and remains a major cause of morbidity and mortality in several developing countries. The global incidence of TB was estimated to be 9.9 million in 2021 (1). Approximately one-fourth of the general population was thought to be infected with MTB, while only 5–10% of these individuals eventually developed active TB during their lifetime (2, 3). This implied that the development of TB involved complex interactions, and bacteria, host genetic variation, and environmental factors played important roles (4). Previous studies have identified various host genes associated with pulmonary TB (PTB) susceptibility; unfortunately, the genetic factors influencing PTB development are not fully understood (5, 6). Therefore, identifying the role of host genetic variations in the development of PTB would facilitate further understanding of the pathogenesis of PTB and guide treatment strategies.

It has been suggested that a successful immune response against MTB infection depends on the activation of CD4+T lymphocytes (7). The Th17 cells, known as novel members of the CD4+T lymphocyte family, can induce interleukin (IL)-21 and IL-23R expression to promote cell proliferation and maturation and secrete several cytokines such as IL-17A, IL-17F, IL-22 (8, 9). Th17 cells play a key role in immunity against PTB, and previous studies have found that reduced Th17 response is related to severe outcomes of MTB infection (10). In addition, the secretion of Th17-related cytokines may contribute to the immune response of the human host against MTB (11). For example, the production of IL-17A can eliminate the primary infection and establish an effective memory response, and IL-17A can augment autophagy in MTB-infected monocytes from individuals with strong immunity against the bacterium (12, 13). A recent study found that the production of IL-17F using an *in vitro* model of human primary cell cultures was stimulated by MTB-Ag and demonstrated that individuals that mount an effective immune response against MTB secreted the highest concentrations of IL-17F (7). This suggested that IL-17F played a protective role in the immune response of the host against mycobacteria.

Several studies have shown that genetic variations in Th17 pathway genes (including *IF-17A*, *IF-17F, IL-21*, and *IL-23R*) are associated with infectious disease susceptibility and clinical manifestations (14, 15). However, the reports of studies on Th17 pathway gene variation and PTB susceptibility have been inconsistent (16). In addition, only a few studies have examined the association between these gene variants and the clinical manifestations of PTB. Therefore, it is important to further evaluate the roles of Th17 pathway gene variations in the pathogenesis of PTB through genetic susceptibility association studies. This study was conducted to investigate the possible roles of the *IL-17A*, *IL-17F*, *IL-21*, and *IL-23R* gene variation in PTB susceptibility and their clinical manifestations.

## Materials and methods

### Study participants

A total of 456 PTB patients were enrolled from Anhui Chest Hospital in this investigation. They included 194 females and 262 males with an average age of 43.37 ± 13.90 years. The diagnosis of PTB was based on the following: suspicious clinical symptoms, chest radiography, sputum and/or bronchoalveolar lavage fluid MTB culture, microscopy for acid-fast bacilli, and the effect of anti-TB treatment. Patients were excluded from this study if they had diseases such as cancer, acquired immunodeficiency syndrome, hepatitis, or immune deficiency. Meanwhile, 466 healthy individuals with the same ethnic background and no history of TB, cancer, and acquired immunodeficiency syndrome were enrolled from the same area as controls. The controls comprised 203 males and 263 females (average age: 45.61 ± 17.68 years), and were asymptomatic with negative sputum smear and culture and had normal chest radiographs.

This study was approved by the ethics committee of Anhui Medical University (20200250), and written informed consent was obtained from every participant before enrolment in the study. We collected peripheral blood samples and relevant data, including demographic characteristics and clinical manifestations (pulmonary infection, leukopenia, fever, drug resistance, drug-induced liver injury (DILI), and sputum smear status) from all the participants.

### Single nucleotide polymorphism (SNP) selection and genotyping

Two approaches were used for SNP selection: literature retrieval and tag SNP selection. Previous studies on the association between the Th17 pathway gene (*IL-17A*, *IL-17F*, *IL-21*, and *IL-23R*) polymorphisms and human diseases were reviewed to identify SNPs related to human disease susceptibility. The genotype data of *IL-17A*, *IL-17F*, *IL-21*, and *IL-23R* in CHB were obtained from Ensembl Genome Browser 85 and CHBS_1000g, and we used the HaploView 4.0 software (Cambridge, MA, USA) to select the tag SNPs, which captured all the common SNPs located in the chromosome locus transcribed into these genes and their flanking 2 000 bp region. In addition, all SNPs included in this study had to meet the following two criteria: minor allele frequency of ≥ 0.05 in CHB and *r^2^* threshold of > 0.8. We finally selected 10 SNPs (*IL-17A* gene rs2275913, rs3748067, rs8193036, rs3819024, *IL-17F* gene rs7741835, rs763780, *IL-21* gene rs907715, rs2055979, *IL-23R* gene rs11805303, and rs7518660) for genotyping.

Peripheral blood was drawn from the medial cubital vein, collected in EDTA-containing tubes, and preserved at a temperature of -20°C. The Flexi Gene-DNA Kit (Qiagen, Valencia, CA) was used to extract genomic DNA from the peripheral blood. With the technical support of the Center for Genetic and Genomic Analysis, Genesky Biotechnologies (Inc., Shanghai), genetic polymorphism was detected using SNPscan technique.

### Statistical analysis

All statistical analyses were conducted using SPSS 23.0 (Armonk, NY: IBM Corp, USA). The Chi-squared (*χ^2^*) test was used to confirm that the genotype distribution of all the SNPs in normal controls was consistent with the Hardy-Weinberg Equilibrium. The frequency distributions of genotypes and alleles in PTB patients and normal controls were compared using the chi-square test (*χ^2^*), and odds ratios with 95% confidence intervals were calculated using logistic regression analysis. The SHEsis software was used to perform Haplotype analysis (17), and the relationship between the Th17 pathway gene variations and susceptibility to PTB under two genetic models (dominant and recessive models) was also examined. A two-tailed *P-*value < 0.05 was considered as statistical significance.

## Results

### Association of Th17 pathway gene polymorphisms with susceptibility to PTB

The allele-genotype frequency distribution of the Th17 pathway gene polymorphisms (*IL-17A* gene rs2275913, rs3748067, rs8193036, rs3819024, *IL-17F* gene rs7741835, rs763780, *IL-21* gene rs907715, rs2055979, *IL-23R* gene rs11805303, and rs7518660) are shown in **Table 1**. The genotype frequencies of these SNPs in normal controls were determined by the Hardy-Weinberg Equilibrium test, and we found that all SNPs reached genetic equilibrium. In *IL-23R* gene, the rs11805303 CC genotype frequency was significantly lower in the PTB patients than in the controls (CC vs. TT: *P* = 0.021), and the patients carrying the C allele had lower susceptibility to PTB than the T allele carriers (C vs. T: *P* = 0.024). In addition, the rs11805303 variant was associated with the reduced risk of PTB under the recessive model (CC vs. CT+TT: *P* = 0.037).

**Table 1:** Association between Th17 pathway genes polymorphisms and PTB susceptibility.

SNP	Analyze model		PTB patients	Controls	*P* value	OR (95% CI)
*IL-17A*						
rs2275913	Genotypes	AA	87 (19.08)	106 (22.75)	0.187	0.783 (0.544,1.126)
		GA	217 (47.59)	215 (46.14)	0.802	0.963 (0.716,1.294)
		GG	152 (33.33)	145 (31.12)	Reference	
	Alleles	A	391 (42.87)	427 (46.82)	0.203	0.936 (0.845,1.037)
		G	521 (57.13)	505 (54.185)	Reference	
	Dominant model	GG	152 (33.33)	145 (31.12)	0.471	1.071 (0.888,1.292)
		GA+AA	304 (66.67)	321 (68.88)	Reference	
	Recessive model	AA	87 (19.08)	106 (22.75)	0.171	0.839 (0.652,1.080)
		GA+GG	369 (80.92)	360 (77.25)	Reference	
rs3748067	Genotypes	TT	8 (1.75)	13 (2.79)	0.296	0.621 (0.254,1.518)
		CT	116 (25.44)	118 (25.32)	0.957	0.992 (0.736,1.336)
		CC	332 (72.81)	335 (71.89)	Reference	
	Alleles	T	132 (14.47)	144 (15.45)	0.557	0.937 (0.753,1.165)
		C	780 (85.53)	788 (84.55)	Reference	
	Dominant model	CC	332 (72.81)	335 (71.89)	0.755	1.013 (0.935,1.097)
		CT+TT	124 (27.19)	131 (28.11)	Reference	
	Recessive model	TT	8 (1.75)	13 (2.79)	0.292	0.629 (0.263,1.503)
		CT+CC	448 (98.25)	453 (97.21)	Reference	
rs8193036	Genotypes	TT	40 (8.77)	38 (8.15)	0.714	1.094 (0.677,1.767)
		CT	187 (41.01)	190 (40.77)	0.870	1.023 (0.780,1.342)
		CC	229 (50.22)	238 (51.07)	Reference	
	Alleles	T	267 (29.28)	266 (28.54)	0.728	1.026 (0.889,1.184)
		C	645 (70.72)	666 (71.46)	Reference	
	Dominant model	CC	229 (50.22)	238 (51.07)	0.795	0.983 (0.866,1.117)
		CT+TT	227 (49.78)	228 (48.93)	Reference	
	Recessive model	TT	40 (8.77)	38 (8.15)	0.736	1.076 (0.703,1.645)
		CT+CC	416 (91.23)	428 (91.85)	Reference	
rs3819024	Genotypes	GG	97 (21.27)	113 (24.25)	0.219	0.798 (0.557,1.144)
		AG	217 (47.59)	221 (47.42)	0.554	0.913 (0.675,1.235)
		AA	142 (31.14)	132 (28.33)	Reference	
	Alleles	G	411 (45.07)	447 (47.96)	0.213	0.940 (0.852,1.036)
		A	501 (54.93)	485 (52.04)	Reference	
	Dominant model	AA	142 (31.14)	132 (28.33)	0.350	1.099 (0.901,1.341)
		AG+GG	314 (68.86)	334 (71.67)	Reference	
	Recessive model	GG	97 (21.27)	113 (24.25)	0.281	0.877 (0.691,1.114)
		AG+AA	359 (78.73)	353 (75.75)	Reference	
*IL-17F*						
rs7741835	Genotypes	TT	42 (9.21)	44 (9.44)	0.859	1.043 (0.656,1.658)
		CT	209 (45.83)	198 (42.49)	0.303	1.153 (0.879,1.513)
		CC	205 (44.96)	224 (48.07)	Reference	
	Alleles	T	293 (32.13)	286 (30.69)	0.505	1.047 (0.915,1.198)
		C	619 (67.87)	646 (69.31)	Reference	
	Dominant model	CC	205 (44.96)	224 (48.07)	0.343	0.935 (0.814,1.074)
		CT+TT	251 (55.04)	242 (51.93)	Reference	
	Recessive model	TT	42 (9.21)	44 (9.44)	0.904	1.003 (0.962,1.045)
		CT+CC	414 (90.79)	422 (90.56)	Reference	
rs763780	Genotypes	CC	4 (0.88)	9 (1.93)	0.193	0.455 (0.139,1.490)
		TC	106 (23.25)	103 (22.1)	0.744	1.053 (0.773,1.434)
		TT	346 (75.88)	354 (75.97)	Reference	
	Alleles	C	114 (12.50)	121 (12.98)	0.756	0.963 (0.758,1.223)
		T	798 (87.50)	811 (87.02)	Reference	
	Dominant model	TT	346 (75.88)	354 (75.97)	0.975	1.004 (0.798,1.262)
		TC+CC	110 (24.12)	112 (24.03)	Reference	
	Recessive model	CC	4 (0.88)	9 (1.93)	0.175	0.454 (0.141,1.464)
		TC+TT	452 (99.12)	457 (98.07)	Reference	
*IL-21*						
rs907715	Genotypes	TT	81 (17.76)	81 (17.38)	0.942	0.986 (0.669,1.453)
		CT	236 (51.75)	248 (53.22)	0.671	0.938 (0.698,1.261)
		CC	139 (30.48)	137 (29.4)	Reference	
	Alleles	T	398 (43.64)	410 (43.99)	0.879	0.992 (0.895,1.100)
		C	514 (56.36)	522 (56.01)	Reference	
	Dominant model	CC	139 (30.48)	137 (29.40)	0.720	1.037 (0.851,1.263)
		CT+TT	317 (69.52)	329 (70.60)	Reference	
	Recessive model	TT	81 (17.76)	81 (17.38)	0.879	0.995 (0.938,1.057)
		CT+CC	375 (82.24)	385 (82.62)	Reference	
rs2055979	Genotypes	AA	73 (16.01)	81 (17.38)	0.597	0.901 (0.613,1.325)
		CA	224 (49.12)	226 (48.50)	0.952	0.991 (0.744,1.321)
		CC	159 (34.87)	159 (34.12)	Reference	
	Alleles	A	370 (40.57)	388 (41.63)	0.643	0.975 (0.795,1.152)
		C	542 (59.43)	544 (58.37)	Reference	
	Dominant model	CC	159 (34.87)	159 (34.12)	0.811	1.022 (0.855,1.221)
		CA+AA	297 (65.13)	307 (65.88)	Reference	
	Recessive model	AA	73 (16.01)	81 (17.38)	0.576	0.921 (0.690,1.229)
		CA+CC	383 (83.99)	385 (82.62)	Reference	
*IL-23R*						
rs11805303	Genotypes	CC	76 (16.67)	103 (22.1)	0.021	0.641 (0.439,0.934)
		CT	228 (50.00)	231 (49.57)	0.308	0.857 (0.637,1.153)
		TT	152 (33.33)	132 (28.33)	Reference	
	Alleles	C	380 (41.67)	437 (46.89)	0.024	0.889 (0.802,0.985)
		T	532 (58.33)	495 (53.11)	Reference	
	Dominant model	TT	152 (33.33)	132 (28.33)	0.100	1.177 (0.969,1.429)
		CT+CC	304 (66.67)	334 (71.67)	Reference	
	Recessive model	CC	76 (16.67)	103 (22.10)	0.037	0.754 (0.577,0.985)
		CT+TT	380 (83.33)	363 (77.90)	Reference	
rs7518660	Genotypes	AA	33 (7.24)	32 (6.87)	0.873	1.043 (0.623,1.746)
		GA	160 (35.09)	168 (36.05)	0.790	0.963 (0.731,1.269)
		GG	263 (57.68)	266 (57.08)	Reference	
	Alleles	A	226 (24.78)	232 (24.89)	0.956	1.001 (0.950,1.055)
		G	686 (75.22)	700 (75.11)	Reference	
	Dominant model	GG	263 (57.68)	266 (57.08)	0.855	0.986 (0.849,1.146)
		GA+AA	193 (42.32)	200 (42.92)	Reference	
	Recessive model	AA	33 (7.24)	32 (6.87)	0.826	1.054 (0.659,1.684)
		GA+GG	423 (92.76)	434 (93.13)	Reference	

The genotype and allele frequencies of the *IL-17A* gene rs2275913, rs3748067, rs8193036, and rs3819024 variants did not affect susceptibility to PTB (all *P* > 0.05). Similarly, our results found no associations between the *IL-17F* gene rs7741835, rs763780, *IL-21* gene rs907715, and rs2055979 variants and PTB susceptibility (all *P* > 0.05).

### Influence of Th17 pathway gene polymorphisms on clinical manifestations of PTB

During PTB development, patients have various clinical manifestations, including fever, drug resistance, DILI, pulmonary infection, and hypoproteinemia, which influence treatment and affect prognosis. These clinical manifestations are generally affected by host genetic variation; hence, this study determined whether the polymorphisms of Th17 pathway genes influence the development of these clinical manifestations (**Table 2**). We found that the AA genotype and A allele carrier of *IL-17A* rs2275913 markedly increased the risk of drug resistance (*P* = 0.001, *P* = 0.003, respectively), and a higher frequency of the rs3748067 TT genotype was associated with drug resistance in PTB patients (*P* = 0.016). On the other hand, the *IL-17A* rs3819024 GG genotype and G allele frequencies were significantly increased in PTB patients with drug resistance relative to the patients without this clinical manifestation (*P* = 0.013, *P* = 0.017, respectively).

**Table 2:** Association between Th17 pathway genes polymorphisms and the clinical manifestations of PTB patients.

SNP	Allele	Clinical features	Group	Genotype n (%)			*P* value	Allele n (%)		*P* value
	(M/m)			MM	Mm	mm		M	m
*IL-17A*										
rs2275913	G/A	fever	+	23 (32.86)	37 (52.86)	10 (14.29)	0.476	83 (59.29)	57 (40.71)	0.575
			–	129 (33.42)	180 (46.63)	77 (19.95)		438 (56.74)	334 (43.26)	
		drug resistance	+	20 (27.40)	27 (36.99)	26 (35.62)	**0.001**	67 (45.89)	79 (54.11)	**0.003**
			–	132 (34.46)	190 (49.61)	61 (15.93)		454 (59.27)	312 (40.73)	
		DILI	+	19 (28.79)	39 (59.09)	8 (12.12)	0.101	77 (58.33)	55 (41.67)	0.762
			–	133 (34.10)	178 (45.64)	79 (20.26)		444 (56.92)	336 (43.08)	
		pulmonary infection	+	30 (36.59)	38 (46.34)	14 (17.07)	0.756	98 (59.76)	66 (40.24)	0.453
			–	122 (32.62)	179 (47.86)	73 (19.52)		423 (56.55)	325 (43.45)	
		hypoproteinemia	+	14 (35.9)	21 (53.85)	4 (10.26)	0.337	49 (62.82)	29 (37.18)	0.288
			–	138 (33.09)	196 (47)	83 (19.9)		472 (56.59)	362 (43.41)	
		leukopenia	+	7 (23.33)	18 (60)	5 (16.67)	0.350	32 (53.33)	28 (46.67)	0.539
			–	145 (34.04)	199 (46.71)	82 (19.25)		489 (57.39)	363 (42.61)	
		sputum smear-positive	+	42 (33.33)	59 (46.83)	25 (19.84)	0.880	143 (56.75)	109 (43.25)	0.791
			–	97 (33.33)	142 (48.8)	52 (17.87)		336 (57.73)	246 (42.27)	
rs3748067	C/T	fever	+	50 (71.43)	19 (27.14)	1 (1.43)	0.920	119 (85)	21 (15)	0.847
			–	282 (73.06)	97 (25.13)	7 (1.81)		661 (85.62)	111 (14.38)	
		drug resistance	+	55 (75.34)	14 (19.18)	4 (5.48)	**0.016**	124 (84.93)	22 (15.07)	0.824
			–	277 (72.32)	102 (26.63)	4 (1.04)		656 (85.64)	110 (14.36)	
		DILI	+	45 (68.18)	21 (31.82)	0 (0)	0.245	111 (84.09)	21 (15.91)	0.612
			–	287 (73.59)	95 (24.36)	8 (2.05)		669 (85.77)	111 (14.23)	
		pulmonary infection	+	61 (74.39)	20 (24.39)	1 (1.22)	0.887	142 (86.59)	22 (13.41)	0.670
			–	271 (72.46)	96 (25.67)	7 (1.87)		638 (85.29)	110 (14.71)	
		hypoproteinemia	+	31 (79.49)	8 (20.51)	0 (0)	0.493	70 (89.74)	8 (10.26)	0.268
			–	301 (72.18)	108 (25.9)	8 (1.92)		710 (85.13)	124 (14.87)	
		leukopenia	+	22 (73.33)	8 (26.67)	0 (0)	0.747	52 (86.67)	8 (13.33)	0.795
			–	310 (72.77)	108 (25.35)	8 (1.88)		728 (85.45)	124 (14.55)	
		sputum smear-positive	+	94 (74.6)	29 (23.02)	3 (2.38)	0.603	217 (86.11)	35 (13.89)	0.787
			–	210 (72.16)	77 (26.46)	4 (1.37)		497 (85.4)	85 (14.6)	
rs8193036	C/T	fever	+	32 (45.71)	31 (44.29)	7 (10)	0.707	95 (67.86)	45 (32.14)	0.418
			–	197 (51.04)	156 (40.41)	33 (8.55)		550 (71.24)	222 (28.76)	
		drug resistance	+	44 (60.27)	23 (31.51)	6 (8.22)	0.158	111 (76.03)	35 (23.97)	0.124
			–	185 (48.3)	164 (42.82)	34 (8.88)		534 (69.71)	232 (30.29)	
		DILI	+	31 (46.97)	29 (43.94)	6 (9.09)	0.847	91 (68.94)	41 (31.06)	0.626
			–	198 (50.77)	158 (40.51)	34 (8.72)		554 (71.03)	226 (28.97)	
		pulmonary infection	+	39 (47.56)	32 (39.02)	11 (13.41)	0.260	110 (67.07)	54 (32.93)	0.257
			–	190 (50.8)	155 (41.44)	29 (7.75)		535 (71.52)	213 (28.48)	
		hypoproteinemia	+	20 (51.28)	14 (35.9)	5 (12.82)	0.583	54 (69.23)	24 (30.77)	0.762
			–	209 (50.12)	173 (41.49)	35 (8.39)		591 (70.86)	243 (29.14)	
		leukopenia	+	17 (56.67)	12 (40)	1 (3.33)	0.507	46 (76.67)	14 (23.33)	0.295
			–	212 (49.77)	175 (41.08)	39 (9.15)		599 (70.31)	253 (29.69)	
		sputum smear-positive	+	66 (52.38)	48 (38.1)	12 (9.52)	0.894	180 (71.43)	72 (28.57)	0.775
			–	146 (50.17)	118 (40.55)	27 (9.28)		410 (70.45)	172 (29.55)	
rs3819024	A/G	fever	+	21 (30)	39 (55.71)	10 (14.29)	0.214	81 (57.86)	59 (42.14)	0.450
			–	121 (31.35)	178 (46.11)	87 (22.54)		420 (54.4)	352 (45.6)	
		drug resistance	+	19 (26.03)	29 (39.73)	25 (34.25)	**0.013**	67 (45.89)	79 (54.11)	**0.017**
			–	123 (32.11)	188 (49.09)	72 (18.8)		434 (56.66)	332 (43.34)	
		DILI	+	17 (25.76)	40 (60.61)	9 (13.64)	0.061	74 (56.06)	58 (43.94)	0.779
			–	125 (32.05)	177 (45.38)	88 (22.56)		427 (54.74)	353 (45.26)	
		pulmonary infection	+	27 (32.93)	38 (46.34)	17 (20.73)	0.928	92 (56.1)	72 (43.9)	0.741
			–	115 (30.75)	179 (47.86)	80 (21.39)		409 (54.68)	339 (45.32)	
		hypoproteinemia	+	12 (30.77)	19 (48.72)	8 (20.51)	0.988	43 (55.13)	35 (44.87)	0.971
			–	130 (31.18)	198 (47.48)	89 (21.34)		458 (54.92)	376 (45.08)	
		leukopenia	+	7 (23.33)	19 (63.33)	4 (13.33)	0.197	33 (55)	27 (45)	0.992
			–	135 (31.69)	198 (46.48)	93 (21.83)		468 (54.93)	384 (45.07)	
		sputum smear-positive	+	37 (29.37)	57 (45.24)	32 (25.4)	0.409	131 (51.98)	121 (48.02)	0.326
			–	90 (30.93)	144 (49.48)	57 (19.59)		324 (55.67)	258 (44.33)	
*IL-17F*										
rs7741835	C/T	fever	+	28 (40)	34 (48.57)	8 (11.43)	0.598	90 (64.29)	50 (35.71)	0.323
			–	177 (45.85)	175 (45.34)	34 (8.81)		529 (68.52)	243 (31.48)	
		drug resistance	+	42 (57.53)	29 (39.73)	2 (2.74)	**0.021**	113 (77.4)	33 (22.6)	**0.007**
			–	163 (42.56)	180 (47)	40 (10.44)		506 (66.06)	260 (33.94)	
		DILI	+	32 (48.48)	28 (42.42)	6 (9.09)	0.814	92 (69.7)	40 (30.3)	0.627
			–	173 (44.36)	181 (46.41)	36 (9.23)		527 (67.56)	253 (32.44)	
		pulmonary infection	+	35 (42.68)	37 (45.12)	10 (12.2)	0.579	107 (65.24)	57 (34.76)	0.426
			–	170 (45.45)	172 (45.99)	32 (8.56)		512 (68.45)	236 (31.55)	
		hypoproteinemia	+	15 (38.46)	17 (43.59)	7 (17.95)	0.137	47 (60.26)	31 (39.74)	0.132
			–	190 (45.56)	192 (46.04)	35 (8.39)		572 (68.59)	262 (31.41)	
		leukopenia	+	11 (36.67)	16 (53.33)	3 (10)	0.636	38 (63.33)	22 (36.67)	0.436
			–	194 (45.54)	193 (45.31)	39 (9.15)		581 (68.19)	271 (31.81)	
		sputum smear-positive	+	57 (45.24)	57 (45.24)	12 (9.52)	0.997	171 (67.86)	81 (32.14)	0.958
			–	132 (45.36)	132 (45.36)	27 (9.28)		396 (68.04)	186 (31.96)	
rs763780	T/C	fever	+	47 (67.14)	22 (31.43)	1 (1.43)	0.174	116 (82.86)	24 (17.14)	0.071
			–	299 (77.46)	84 (21.76)	3 (0.78)		682 (88.34)	90 (11.66)	
		drug resistance	+	59 (80.82)	14 (19.18)	0 (0)	0.436	132 (90.41)	14 (9.59)	0.246
			–	287 (74.93)	92 (24.02)	4 (1.04)		666 (86.95)	100 (13.05)	
		DILI	+	56 (84.85)	10 (15.15)	0 (0)	0.160	122 (92.42)	10 (7.58)	0.064
			–	290 (74.36)	96 (24.62)	4 (1.03)		676 (86.67)	104 (13.33)	
		pulmonary infection	+	55 (67.07)	25 (30.49)	2 (2.44)	0.048	135 (82.32)	29 (17.68)	0.027
			–	291 (77.81)	81 (21.66)	2 (0.53)		663 (88.64)	85 (11.36)	
		hypoproteinemia	+	28 (71.79)	9 (23.08)	2 (5.13)	**0.012**	65 (83.33)	13 (16.67)	0.245
			–	318 (76.26)	97 (23.26)	2 (0.48)		733 (87.89)	101 (12.11)	
		leukopenia	+	25 (83.33)	5 (16.67)	0 (0)	0.573	55 (91.67)	5 (8.33)	0.313
			–	321 (75.35)	101 (23.71)	4 (0.94)		743 (87.21)	109 (12.79)	
		sputum smear-positive	+	93 (73.81)	31 (24.6)	2 (1.59)	0.590	217 (86.11)	35 (13.89)	0.414
			–	224 (76.98)	65 (22.34)	2 (0.69)		513 (88.14)	69 (11.86)	
*IL-21*										
rs907715	C/T	fever	+	17 (24.29)	43 (61.43)	10 (14.29)	0.212	77 (55)	63 (45)	0.724
			–	122 (31.61)	193 (50)	71 (18.39)		437 (56.61)	335 (43.39)	
		drug resistance	+	21 (28.77)	43 (58.9)	9 (12.33)	0.303	85 (58.22)	61 (41.78)	0.621
			–	118 (30.81)	193 (50.39)	72 (18.8)		429 (56.01)	337 (43.99)	
		DILI	+	19 (28.79)	38 (57.58)	9 (13.64)	0.517	76 (57.58)	56 (42.42)	0.761
			–	120 (30.77)	198 (50.77)	72 (18.46)		438 (56.15)	342 (43.85)	
		pulmonary infection	+	25 (30.49)	39 (47.56)	18 (21.95)	0.515	89 (54.27)	75 (45.73)	0.551
			–	114 (30.48)	197 (52.67)	63 (16.84)		425 (56.82)	323 (43.18)	
		hypoproteinemia	+	10 (25.64)	25 (64.1)	4 (10.26)	0.230	45 (57.69)	33 (42.31)	0.804
			–	129 (30.94)	211 (50.6)	77 (18.47)		469 (56.24)	365 (43.76)	
		leukopenia	+	5 (16.67)	17 (56.67)	8 (26.67)	0.166	27 (45)	33 (55)	0.066
			–	134 (31.46)	219 (51.41)	73 (17.14)		487 (57.16)	365 (42.84)	
		sputum smear-positive	+	39 (30.95)	67 (53.17)	20 (15.87)	0.885	145 (57.54)	107 (42.46)	0.717
			–	88 (30.24)	151 (51.89)	52 (17.87)		327 (56.19)	255 (43.81)	
rs2055979	C/A	fever	+	20 (28.57)	42 (60)	8 (11.43)	0.134	82 (58.57)	58 (41.43)	0.822
			–	139 (36.01)	182 (47.15)	65 (16.84)		460 (59.59)	312 (40.41)	
		drug resistance	+	17 (23.29)	35 (47.95)	21 (28.77)	0.147	69 (47.26)	77 (52.74)	0.072
			–	56 (14.62)	189 (49.35)	138 (36.03)		301 (39.3)	465 (60.7)	
		DILI	+	25 (37.88)	33 (50.00)	8 (12.12)	**＜0.001**	83 (62.88)	49 (37.12)	**＜0.001**
			–	65 (16.67)	191 (48.97)	134 (34.36)		321 (41.15)	459 (58.85)	
		pulmonary infection	+	29 (35.37)	39 (47.56)	14 (17.07)	0.938	97 (59.15)	67 (40.85)	0.935
			–	130 (34.76)	185 (49.47)	59 (15.78)		445 (59.49)	303 (40.51)	
		hypoproteinemia	+	10 (25.64)	22 (56.41)	7 (17.95)	0.449	42 (53.85)	36 (46.15)	0.294
			–	149 (35.73)	202 (48.44)	66 (15.83)		500 (59.95)	334 (40.05)	
		leukopenia	+	14 (46.67)	14 (46.67)	2 (6.67)	0.215	42 (70)	18 (30)	0.084
			–	145 (34.04)	210 (49.3)	71 (16.67)		500 (58.69)	352 (41.31)	
		sputum smear-positive	+	37 (29.37)	69 (54.76)	20 (15.87)	0.362	143 (56.75)	109 (43.25)	0.385
			–	105 (36.08)	139 (47.77)	47 (16.15)		349 (59.97)	233 (40.03)	
*IL-23R*										
rs11805303	T/C	fever	+	25 (35.71)	36 (51.43)	9 (12.86)	0.639	86 (61.43)	54 (38.57)	0.419
			–	127 (32.9)	192 (49.74)	67 (17.36)		446 (57.77)	326 (42.23)	
		drug resistance	+	21 (28.77)	39 (53.42)	13 (17.81)	0.665	81 (55.48)	65 (44.52)	0.445
			–	131 (34.2)	189 (49.35)	63 (16.45)		451 (58.88)	315 (41.12)	
		DILI	+	24 (36.36)	29 (43.94)	13 (19.7)	0.548	77 (58.33)	55 (41.67)	1.000
			–	128 (32.82)	199 (51.03)	63 (16.15)		455 (58.33)	325 (41.67)	
		pulmonary infection	+	35 (42.68)	35 (42.68)	12 (14.63)	0.139	105 (64.02)	59 (35.98)	0.103
			–	117 (31.28)	193 (51.6)	64 (17.11)		427 (57.09)	321 (42.91)	
		hypoproteinemia	+	13 (33.33)	16 (41.03)	10 (25.64)	0.253	42 (53.85)	36 (46.15)	0.401
			–	139 (33.33)	212 (50.84)	66 (15.83)		490 (58.75)	344 (41.25)	
		leukopenia	+	12 (40)	13 (43.33)	5 (16.67)	0.700	37 (61.67)	23 (38.33)	0.588
			–	140 (32.86)	215 (50.47)	71 (16.67)		495 (58.1)	357 (41.9)	
		sputum smear-positive	+	44 (34.92)	65 (51.59)	17 (13.49)	0.364	153 (60.71)	99 (39.29)	0.261
			–	94 (32.3)	141 (48.45)	56 (19.24)		329 (56.53)	253 (43.47)	
rs7518660	G/A	fever	+	43 (61.43)	25 (35.71)	2 (2.86)	0.301	111 (79.29)	29 (20.71)	0.226
			–	220 (56.99)	135 (34.97)	31 (8.03)		575 (74.48)	197 (25.52)	
		drug resistance	+	39 (53.42)	31 (42.47)	3 (4.11)	0.247	109 (74.66)	37 (25.34)	0.864
			–	224 (58.49)	129 (33.68)	30 (7.83)		577 (75.33)	189 (24.67)	
		DILI	+	39 (59.09)	22 (33.33)	5 (7.58)	0.948	100 (75.76)	32 (24.24)	0.877
			–	224 (57.44)	138 (35.38)	28 (7.18)		586 (75.13)	194 (24.87)	
		pulmonary infection	+	53 (64.63)	24 (29.27)	5 (6.1)	0.371	130 (79.27)	34 (20.73)	0.185
			–	210 (56.15)	136 (36.36)	28 (7.49)		556 (74.33)	192 (25.67)	
		hypoproteinemia	+	18 (46.15)	17 (43.59)	4 (10.26)	0.302	53 (67.95)	25 (32.05)	0.120
			–	245 (58.75)	143 (34.29)	29 (6.95)		633 (75.9)	201 (24.1)	
		leukopenia	+	18 (60)	10 (33.33)	2 (6.67)	0.964	46 (76.67)	14 (23.33)	0.788
			–	245 (57.51)	150 (35.21)	31 (7.28)		640 (75.12)	212 (24.88)	
		sputum smear-positive	+	68 (53.97)	48 (38.10)	10 (7.94)	0.609	184 (73.02)	68 (26.98)	0.399
			–	172 (59.11)	97 (33.33)	22 (7.56)		441 (75.77)	141 (24.23)	

Bold value means P < 0.05.

For the *IL-17F* gene, the rs7741835 TT genotype and T allele frequencies in PTB patients with drug resistance were significantly lower than those in the patients without drug resistance (*P* = 0.021, *P* = 0.007, respectively), and the rs763780 C allele frequency was increased in PTB patients with hypoproteinemia (*P* = 0.012). Regarding the *IL-21* gene, PTB patients carrying the TT genotype and T allele of rs2055979 showed reduced susceptibility to DILI (*P* < 0.001, *P* < 0.001, respectively). However, the *IL-23R* rs11805303 and rs7518660 variants had no relationship with the clinical manifestations of PTB patients (all *P* > 0.05).

### Haplotype analysis

We detected the main haplotypes of the Th17 pathway genes (*IL-17A*, *IL-17F*, *IL-21*, *IL-23R*) determined by the SHEsis software. The main haplotypes we eventually detected were as follows: *IL-17A* ACCA, GCCA, GCCG, GCTA, GTCA, GTTA, *IL-17F* CT, TC, TT, *IL-21* CA, CC, TC*, IL-23R* CA, CG, and TG.

The haplotype distributions in the PTB patients and controls are summarized in **Table 3**. The results suggested that the *IL-23R* CG haplotype frequency was significantly lower in PTB patients than in normal controls (*P* = 0.005), while the TG haplotype frequency was significantly higher (*P* = 0.022). However, there were no significant associations between the *IL-17A, IL-17F*, and *IL-21* haplotype frequencies and PTB risk.

**Table 3:** Haplotype analysis of Th17 pathway genes in PTB patients and controls.

Haplotype	PTB [n (%)]	Controls [n (%)]	*P* value	*OR* (95% CI)
*IL-17A* rs2275913-rs3748067-rs8193036-rs3819024				
ACCA	343.98 (37.7)	392.83 (42.1)	0.099	0.852 (0.704,1.031)
GCCA	190.90 (20.6)	182.67 (19.6)	0.357	1.114 (0.886,1.400)
GCCG	32.66 (3.6)	26.37 (2.8)	0.322	1.301 (0.897,1.454)
GCTA	167.17 (18.3)	156.23 (16.8)	0.282	1.142 (0.897,1.454)
GTCA	44.03 (4.8)	35.76 (3.8)	0.257	1.297 (0.826,2.038)
GTTA	73.73 (8.1)	93.73 (10.1)	0.177	0.802 (0.582,1.105)
*IL-17F* rs7741835-rs763780				
CT	612.64 (67.2)	642.32 (68.9)	0.477	0.931 (0.765,1.134)
TC	107.64 (11.8)	117.32 (12.6)	0.623	0.932 (0.705,1.233)
TT	185.36 (20.3)	168.68 (18.1)	0.213	1.159 (0.919,1.462)
*IL-21* rs907715- rs2055979				
CA	369.99 (40.6)	388.00 (41.6)	0.643	0.957 (0.795,1.152)
CC	144.01 (15.8)	134.00 (14.4)	0.397	1.117 (0.865,1.441)
TC	397.99 (43.6)	410.00 (44.0)	0.879	0.986 (0.820,1.185)
*IL-23R* rs11805303- rs7518660				
CA	221.17 (24.3)	225.62 (24.2)	0.998	1.00 (0.808,1.238)
CG	158.83 (17.4)	211.38 (22.7)	0.005	0.717 (0.570,0.903)
TG	527.17 (57.8)	488.62 (52.4)	0.022	1.241 (1.032,1.492)

frequency < 0.03 in both controls & PTB patients has been dropped.

## Discussion

PTB is a major public health problem but the factors underlying the human immune response to mycobacterium are still largely unknown. Identifying prospective genetic biomarkers of PTB is important because it may improve our understanding of its pathogenesis and facilitate early diagnosis and prompt clinical treatment. It is widely known that Th17 cells are involved in adaptive immunity against MTB, and Th17-related cytokines are essential regulators of anti-TB immune responses (18, 19). The contribution of Th17 pathway gene polymorphisms to PTB has been studied, with some studies reporting conflicting results. For example, several reports by studies on *IL-17A* rs2275913, rs3748067, rs3819024, and PTB susceptibility are inconsistent (20, 21). Hence, our study verified the associations between selected SNPs and PTB risk. In addition, the roles of *IL-17F, IL-21*, and *IL-23R* gene variations in the development of PTB among the Chinese Han population have been poorly studied, and we explored the genetic polymorphisms of *IL-17F* rs7741835, rs763780*, IL-21* rs907715, rs2055979*, IL-23R* rs11805303, and rs7518660 and their associations with PTB.

Cytokine secretion is induced by the interaction between different immune cells and bacteria, and IL-17 acts as a pro-inflammatory cytokine by recruiting granulocytes to the sites of infection (22, 23). IL-17A and IL-17F, which belong to the IL-17 family, have similar biological functions and can induce target cells to produce multiple inflammatory cytokines, metalloproteinases, and chemokines, resulting in neutrophil recruitment, activation, and exudation to trigger inflammation (24). Previous studies have demonstrated that the IL-17A concentration increases in TB and is associated with its severity. The IL-17A concentration increased in mouse models and human PBMC cultures *in vitro* after stimulation with MTB in previous studies (24–26). In another study, the authors found that IL-17F secretion increased in the population with an effective immune response against MTB (7). A recent meta-analysis suggested that *IL-17A* rs2275913 polymorphisms may be associated with a reduced risk of PTB in Caucasians, and rs3748067 polymorphism was considered a risk factor for PTB in Asians (21). On the other hand, *IL-17A* rs3819024 and *IL-17F* rs763780 polymorphisms did not influence PTB susceptibility (21). As a functional SNP of *IL-17A*, Wang et al. found that rs8193036 could regulate gene expressions by influencing the binding activity of transcription factors, and rs8193036 T frequency was associated with active PTB (27). However, another study described no relationship between rs8193036 and PTB risk (28). In this study, we did not find any effects of *IL-17A* rs2275913, rs3748067, rs8193036, rs3819024, *IL-17F* rs7741835, and rs763780 polymorphisms on PTB susceptibility. This was consistent with the results of several previous studies (28, 29) and helped improve our understanding of the roles of *IL-17A* and *IL-17F* gene variations in PTB development. Our results were different from those of some studies due to the different sample sizes, ethnicities, genotyping methods, and study design, among others (27, 30). Hence, studies with larger samples and multiple ethnicities are needed.

IL-23 is a key proinflammatory cytokine in the innate and adaptive immune system that was required for long-term control of MTB, and the activity of this cytokine is mediated by its binding to the IL23R complex (31, 32). An important function of IL-23 mediated by the IL-23R complex is to promote the differentiation of T-cells to Th17, thereby increasing the release of other cytokines such as IL-17 and TNF, which are critical for the progression of PTB (12, 33). Several studies have confirmed that several SNPs of the *IL-23R* gene are associated with susceptibility to infectious disease, such as HBeAg-positive chronic hepatitis B (24). The study by Jiang et al. revealed that *IL-23R* rs7518660 was associated with PTB in Chinese Uygurs (34). In contrast, our study did not find a statistical association between rs7518660 and PTB susceptibility in the Chinese Han population. These inconsistent results were largely due to ethnic differences, and more studies were needed to confirm these results. In this study, we also found that the rs11805303 CC genotype and C allele of *IL-23R* were related to PTB. In addition, two haplotype frequencies were also abnormal in patients with PTB; this result supported the hypothesis that the *IL-23R* rs11805303 variant influenced susceptibility to PTB. Previous studies have shown that the IL-21 signaling pathway plays an important role in T-cell response to MTB infection by enhancing CD8+T cell activation and promoting T-cell accumulation in the lung and secretion of T-cell cytokines (35, 36). The level of expression of IL-21 in the peripheral blood of PTB patients also significantly decreases (35). Previous studies have analyzed the association between *IL-21* gene variation and the risk of infectious diseases (37, 38), but its role in PTB has not been explored. This is the first study to analyze the associations between *IL-21* gene rs907715 and rs2055979 polymorphisms and PTB; however, no significant association was found.

Studies have confirmed that host gene variations influence the clinical manifestations and prognosis of PTB patients. Wang et al. found that *IL-17A* rs3819024 and *IL-17F* rs763780 are weakly associated with the prognosis of PTB (27). Our previous study revealed that the *CYP27A1* rs17470271 and rs933994 T alleles were significantly associated with leukopenia and drug resistance in PTB patients, respectively (39). In this study, we provided evidence of the associations between multiple SNPs (*IL-17A* rs2275913, rs3748067, rs3819024, and *IL-17F* rs7741835) and drug resistance in patients with PTB. The *IL-17F* rs763780 and *IL-21* rs2055979 variants were associated with the development of hypoproteinemia and DILI in PTB patients, respectively. Based on these findings, it is reasonable to assume that these SNPs can be used to predict several clinical manifestations of PTB patients, which, in turn, will guide therapeutic schedules for patients.

In summary, this study suggests that *IL-23R* rs11805303 polymorphisms are associated with a decreased susceptibility to PTB, and *IL-17A*, *IL-17F*, and *IL-21* gene polymorphisms do not involve the genetic background of PTB in Chinese. We found genetic evidence of significant relationships between *IL-17A*, *IL-17F*, and *IL-21* genetic variations and several clinical manifestations, including drug resistance and hypoproteinemia in PTB patients. Some limitations of this study need to be considered. Firstly, the possible influence of some confounding factors, such as environmental factors and treatment regimen, in this study was not excluded. Secondly, the sample was relatively small, and larger samples should be used in future studies. Further studies with larger sample sizes that involve different ethnic groups should be conducted to further reveal the roles of Th17 pathway genes in PTB development.

## Data availability statement

The original contributions presented in the study are included in the article. Further inquiries can be directed to the corresponding authors.

## Ethics statement

This study was approved by the ethics committee of Anhui Medical University (20200250). The patients/participants provided their written informed consent to participate in this study.

## Author contributions

T-PZ and H-FP designed the study. H-ML conducted the experiment. L-JW performed the statistical analyses. L-JW and QH participated in sample collection. H-ML drafted the manuscript. T-PZ and H-FP contributed to the manuscript revision. All authors approved the final submitted version.

## Funding

This work was supported by grants from the National Natural Science Foundation of China (82003515).

## Conflict of interest

The authors declare that the research was conducted in the absence of any commercial or financial relationships that could be construed as a potential conflict of interest.

## Publisher’s note

All claims expressed in this article are solely those of the authors and do not necessarily represent those of their affiliated organizations, or those of the publisher, the editors and the reviewers. Any product that may be evaluated in this article, or claim that may be made by its manufacturer, is not guaranteed or endorsed by the publisher.
